# Editorial: Applications of AI, machine learning, computational medicine, and bioinformatics in respiratory pharmacology

**DOI:** 10.3389/fphar.2025.1602941

**Published:** 2025-06-11

**Authors:** Muhammad Ashad Kabir, Paolo Montuschi, Kuang-Ming Liao, Mojtaba Amani

**Affiliations:** ^1^ School of Computing, Mathematics and Engineering, Charles Sturt University, Bathurst, NSW, Australia; ^2^ National Heart and Lung Institute, Faculty of Medicine, Imperial College of Science, Technology and Medicine, London, United Kingdom; ^3^ Pharmacology, Faculty of Medicine, Catholic University of the Sacred Heart, Rome, Italy; ^4^ Department of Internal Medicine, Chi Mei Medical Center, Chiali, Taiwan; ^5^ Department of Nursing, Min-Hwei Junior College of Health Care Management, Tainan, Taiwan; ^6^ Lung Disease Research Center, Ardabil University of Medical Sciences, Ardabil, Iran; ^7^ Department of Medicinal Chemistry, School of Pharmacy, Ardabil University of Medical Sciences, Ardabil, Iran

**Keywords:** artificial intelligence, bioinformatics, machine learning, computational medicine, respiratory pharmacology

Alvin Toffler, in his wave theory, has described three societies in the concept of waves as agricultural society, industrial society, and post-industrial society, AKA Information Age. The information age led to an explosion in data and communication efficiency. These achievements were made possible by reduced prices and fast data acquisition. For example, next-generation sequencing technology can provide the whole genome sequence in just a few hours with affordable prices as low as $100–$200 per genome. Similar data explosions happened in medicine, pharmacology, and other fields. The increase in the volume of data achieved by high-throughput methods is demanding to use a combination of technologies, programming frameworks, statistical methods, and computational methods to manage, classify, find patterns, analyze, interpret biological data, and finally answer the questions of researchers in the medical field. Bioinformatics, computational medicine, systems biology, and computational biology have emerged from these demands.

It seems that we are encountering the fourth wave by developing Artificial Intelligence (AI). Like any previous waves, AI is changing many aspects of human life. AI is not just a tool for problem-solving and decision-making, but it also influences ethics, beliefs, and other human and art sciences. It is expected that some researchers in the AI field focus on ethical AI development and the impact of AI on society.

AI involves a wide range of techniques and approaches, including machine learning, deep learning, natural language processing, computer vision, and robotics. AI has significant applications across various fields, including healthcare and computational medicine, bioinformatics, finance, transportation, education, and many others, and continues to be an active area of research and development.

Bioinformatics and computational medicine utilize AI and its subfields as beneficial tools to solve relevant Research Topic. AI and its subfields have numerous applications in respiratory medicine, such as diagnosing and treating lung diseases, evaluating lung images, pulmonary function test interpretation, monitoring and management of patients, and predicting outcomes.

The application of AI in bioinformatics and computational medicine has the potential to transform respiratory pharmacology in the future by identifying common mechanisms and biomarkers, better understanding the respiratory microbiome and its role in respiratory diseases, predicting drug-drug interactions, and suggesting potential drug candidates for respiratory diseases.

Given the narrow Research Topic of this Research Topic, we were expecting to receive fewer manuscripts. Meanwhile, it was a challenge for us to explore how common AI, machine learning, and computational sciences are in a very specific Research Topic, such as respiratory pharmacology. This expectation did not compel us to ease the reviewing process, and we did not accept many high-quality and valuable manuscripts simply because they did not align with this Research Topic’s scope. Ultimately, we received 22 manuscripts, of which four were accepted and published.

In the study, “A real-world study of antifibrotic drugs-related adverse events based on the United States Food and Drug Administration adverse event reporting system and VigiAccess databases”, the authors analyzed the adverse events and adverse drug reactions associated with pirfenidone and nintedanib, the drugs used in idiopathic pulmonary fibrosis. They mined the reports from the FAERS and VigiAccess databases using the OpenVigil 2.1 online tool.

In the study “Elucidating shared biomarkers in gastroesophageal reflux disease and idiopathic pulmonary fibrosis: insights into novel therapeutic targets and the role of *angelicae sinensis* radix”, the authors sought to elucidate the causal connection and potential mechanisms underlying the coexistence of GERD and IPF. They used Mendelian Randomization to uncover the link between IPF and GERD, SNPs and microarray data to identify potential targets and mechanisms related to IPF in GERD, and network pharmacology and molecular docking to explore the targets and efficacy of *Angelicae sinensis* radix in treating GERD-related IPF.

In the study “Therapeutic targets for lung cancer: genome-wide Mendelian randomization and colocalization analyses,” the authors employed genome-wide Mendelian randomization and colocalization analyses to identify potential therapeutic targets for lung cancer. Their findings suggest that certain genetic variants are associated with lung cancer risk, highlighting new avenues for targeted therapies. These insights contribute to a deeper understanding of lung cancer pathogenesis and may inform future drug development strategies.

In the study, “Development of an algorithm to identify small cell lung cancer patients in claims databases,” the authors address a critical challenge in oncology research: the accurate identification of small cell lung cancer (SCLC) patients within administrative claims databases. The current International Classification of Diseases, 10th Revision (ICD-10) coding system lacks the specificity to distinguish between SCLC and non-small cell lung cancer (NSCLC), complicating efforts to study treatment patterns, outcomes, and healthcare utilization specific to SCLC. To overcome this limitation, the authors developed an algorithm leveraging treatment patterns, particularly the administration of etoposide—a chemotherapy agent predominantly used in SCLC treatment. They validated this algorithm using data from the Surveillance, Epidemiology, and End Results (SEER) program linked with Medicare claims, employing SEER’s histology codes as the gold standard for cancer type classification.

The significance of this study lies in its potential to enhance the accuracy of real-world evidence studies focusing on SCLC. By providing a validated tool to accurately identify SCLC patients, the algorithm enables more precise analyses of treatment patterns, patient outcomes, and healthcare resource utilization specific to this aggressive cancer subtype. This advancement is particularly important given the evolving treatment landscape of SCLC and the need for robust real-world data to inform clinical and policy decisions.

We would like to thank those who helped us in handling this Research Topic, especially reviewers. We hope this Research Topic provides valuable insights into the evolving role of AI in respiratory pharmacology and inspires further research in this exciting interdisciplinary field.

